# Self-identified barriers to health services among migrants 50 years of age or older: population-based survey study of Russian speakers in Finland

**DOI:** 10.1186/s12913-024-10728-3

**Published:** 2024-02-27

**Authors:** Nuriiar Safarov, Laura Kemppainen, Sirpa Wrede, Anne Kouvonen

**Affiliations:** 1 Faculty of Social Sciences, University of Helsinki, Helsinki, Finland; 2 Centre for Public Health, Queen’s University Belfast, Belfast, Northern Ireland

**Keywords:** Older migrants, Health services, Self-identified barriers, Language, Discrimination, Migrants, Survey study

## Abstract

**Background:**

The compounded effect of a migratory background and ageing increases the risk of unequal medical treatment opportunities. The aim of this article is to investigate the social determinants of barriers to health services.

**Methods:**

The study uses population-based survey data of Russian-speaking migrants (50 + years) residing in Finland (*n* = 1082, 57% of men, mean age 63 years). Multiple correspondence analysis was performed as a dimension reduction procedure on six barriers to health services. Multiple ordinary least-squares linear regression was used for the predicted score of the barriers as an outcome variable.

**Results:**

Most of the sociodemographic characteristics were not associated with barriers to health services, except gender, as women tended to face more disadvantages. Migration-related factors, such as the need for interpreters for health services and experienced discrimination, were associated with an increased likelihood of reporting barriers to health services. Using the internet as a primary source of health information was associated with more access barriers to health services.

**Conclusions:**

Migrants 50 years of age or older face multiple barriers to health services. Given that the healthcare needs increase with age, addressing this issue becomes crucial, necessitating improved access to health services for older migrants.

## Background

Equal access to health services for migrants has been recognised as a global challenge [1]. Migrants report more unmet health needs compared to the majority population in various European countries, including Finland [2–4]. With ageing, the risk of health problems as well as healthcare needs increase [5]. The combined effect of age and minority status may place older migrants at a double disadvantage regarding their health [6, 7]. Previous studies from Europe and North America have demonstrated that migrants commonly have more difficulties accessing health services than the general population [4, 8], but there is a significant gap in research on older migrants. Reaching this population in representative surveys is challenging, leading most existing research to rely on qualitative or small sample data [9].

Nordic countries are renowned for their universalistic welfare systems, which are believed to provide equal rights and access to essential services, including healthcare, regardless of citizenship status or nationality. Nevertheless, prior research conducted in Nordic countries has indicated that older migrants experience poorer health and functional ability [10] and utilise health services less frequently [11] compared to the general older population. However, the underlying factors contributing to these disparities remain underexplored. It is particularly important to investigate these challenges in Finland, as the OECD has previously classified the Finnish healthcare system as one of the most inequitable among the developed nations [12, 13].

Earlier reviews have found that, in accessing and utilizing healthcare, individuals with a migration background can face various challenges, encompassing both patient-related and systemic issues. Personal challenges include limited resources, lack of knowledge, past experiences influencing perceptions of care quality, acceptance of care, as well as cultural, emotional, and attitudinal expectations [1, 4, 8]. On the systemic side, previous studies in Finland and elsewhere in Europe have highlighted barriers to healthcare services such as accessibility (including e.g. complex healthcare structure, transportation problems, language barriers), barriers related to culturally unsafe care, as well as, long waiting lists, high costs of services, and discrimination [2, 14, 15].

However, only a few studies have distinguished self-identified barriers to health services and investigated which social determinants are associated with reporting more barriers. As a result, the studies identified only potential risks that migrant populations may face in accessing health services. To address this gap, this article focuses on the analysis of the prevalence of self-reported barriers and of the fundamental reasons behind them. The studied barriers include circumstances related to the healthcare system, such as long waiting times, poor transport connections, high price of services, impolite or poor service, language difficulties, and complicated structure of services.

### Social determinants of access to health services

Studies in Europe have previously recognised the vast importance of the sociodemographic characteristics of working age migrants for health services access and use [4, 16]. The inability to afford primary health services costs can result in postponement of care or unmet healthcare needs [8, 17]. The problem of inability to pay for services is less prevalent in countries with universal access to healthcare, nevertheless systematic inequalities have been observed in access to services that require co-payments, such as dental care [18].

Previous research has documented an array of predictors related to migratory background. Recent evidence suggests that citizenship status constitutes a major obstacle for working-age migrants accessing health services in many countries, especially in those without universal coverage [1]. Poor skills in local languages and the use of interpreters have been found to be associated with poorer quality of received care and access to health services in European countries, including Finland [1, 9, 19, 20]. These findings encompassed both working age and older migrants. Earlier experiences of ethnic or racial discrimination and fears of discrimination have been associated with the reduced access and use of health services of working-age migrants [4, 21–23].

In their systematic review, Ahmadinia et al. [24] found that working-age migrants face disproportionate difficulties in accessing health information in many European countries. **Information-related** determinants such as health literacy and difficulties with health information seeking diminished access to health services for both working age and older migrants [4, 25]. Healthcare is largely entangled in internet use in Nordic countries, as access to health services usually involves the use of online applications for accessing and seeking health information, booking appointments, and renewing prescriptions. Previous research in Finland has pointed to the inequality of access to health services due to difficulties in using digital health and social care services, or the lack of an electronic identification method [26–28].

In sum, existing studies have identified associations of sociodemographic characteristics, migratory background, and health information seeking practices with access to and use of health services among migrants mainly of working age. However, very few studies have investigated this issue in older age groups of migrants specifically [9], and to the best of our knowledge, there are no such previous representative studies conducted in the Nordic countries. Due to the clear evidence from existing research that highlights the unequal access to health services for working-age migrants, it becomes crucial to investigate the obstacles faced by older migrants when trying to access health services. It is particularly important to address this gap, given the combined impact of factors related to aging and migration.

### The Finnish context

The Finnish healthcare system is based on the principle of universalism, which states that every resident has a right to adequate health services without financial hardship and regardless of citizenship [29]. In the case of asylum seekers, the reception centres provide healthcare for them under the supervision of the Finnish Immigration Service [30].

The Finnish healthcare system is based on three parallel channels. Public healthcare is at the core of the system and is mostly tax funded. Other channels are private services, and occupational healthcare, which cover employed people, but its scope varies [31]. The Finnish social and health insurance system includes all residents and reimburses some prescription medicine and a small part of the costs from using private health services [29]. Finland spends less on health than many other EU countries, and in the current healthcare system, out-of-pocket payments account for one fifth of expenditures [31].

The main problems related to unequal access to health services among different population groups in Finland are relatively high out-of-pocket expenditures for health and social care services and medication, spatial accessibility disparities due to small but geographically dispersed populations, and uneven distribution of resources, especially regarding primary and elderly care, which has resulted in long waiting times [29, 32]. In particular, older people and those in low socioeconomic positions are at risk of unequal access to health services [29]. In the general population, older age groups report geographical distance and high out-of-pocket payments as hindering their access to health services more often than in younger cohorts [33]. Working-age migrants have been shown to use health services less often than the general population [20, 34]. They have also reported higher level of unmet healthcare needs and dissatisfaction with the treatment they had received [2, 3].

### The present study

This article delves into the systemic barriers that migrants 50 years of age or older identify as impeding their ability to obtain necessary healthcare services. The aim of the study is to examine the social determinants of self-identified barriers to accessing health services.

The research questions are as follows:

1. Which sociodemographic factors are associated with reporting more barriers to health services among migrants 50 years of age or older?
2. Which factors related to migratory background increase the likelihood of reporting barriers to health services?
3. How are information-seeking practices associated with an increased likelihood of facing barriers to health services?
4. How do those associations differ for two studied age groups, i.e., 50-64-year-olds and 65 + year-olds?

## Methods

### Data

This article draws on a population-based survey study of Russian-speaking migrants in Finland. There were a total of 93,535 Russian-speaking migrants in Finland at the end of 2022, comprising the largest foreign language group of the country, and approximately 30% of them are 50 years of age and older [35]. The data from the survey ‘Care, Health and Ageing of Russian-speaking Minority’ (CHARM) were used for the analyses [28, 36, 37]. CHARM is a representative study of Russian-speaking people 50 years of age and older, permanently and legally residing in Finland. The data were collected in 2019. The survey was sent to people who had Russian registered as their first language. A random sample of 3000 people was obtained from the Digital and Population Data Services Agency. We received 1082 responses (36% response rate). The participants received a letter by post that asked them to answer the questionnaire online or on paper in Russian or Finnish. The sample was stratified by gender. The information from national registers (sex, age, income, pensions, unemployment, and region of residence) was used to adjust for nonresponse bias and compute the weights.

### Dependent variable

The dependent variable is based on the item asking whether several circumstances made it difficult for participants to access health services in Finland during the past 12 months (0 = No, 1 = Yes). Participants had to answer all of the following options: (1) Long waiting lists; (2) Poor transport connections to the place of healthcare service (health centre or hospital); (3) High price of services; (4) Impolite or poor service at the appointment; (5) Language difficulties (for example during the appointment, in phone or using the online services); (6) Complicated structure of the healthcare system in Finland, (7) Access to health services was hindered by another reason, what? The last option was excluded due to many missing values.

### Covariates

The models are adjusted for the self-rated health and service use variables that can reduce or increase the possible effect of service needs on the number of barriers faced. Self-rated health was measured by the question ‘In general, would you say your health now is…’ with 5 answers: good, fairly good, average, fairly poor, and poor [38, 39]. For the analysis, it was dichotomised, 0 = good or fairly good, 1 = average, fairly poor, or poor. Service use was measured by the question ‘How often have you used the following services in Finland during the past 12 months’, with three sub-questions: appointment with a physician or a nurse in the local health centre, appointment with a physician or a nurse in a private medical centre, and appointment with an occupational health physician or nurse. The indicator of the services use was dichotomised for the analysis, with 0 = used services once or have not used any services at all, 1 = used twice or more often at least one of those three services.

### Independent variables

Independent variables include age (0 = 50–64, 1 = 65 and above), gender (0 = woman, 1 = man), education obtained in the country of origin (1 = general, none, or missing, 2 = vocational, 3 = higher), education in Finland (0 = no education, 1 = some education), region of residence (0 = not in the capital region, 1 = in the capital region), marital status (0 = divorced, widowed, or not married, 1 = married or cohabiting), Finnish citizenship (0 = no, 1 = yes), and employment status (1 = employed or self-employed, 2 = receiving disability or statutory pension, 3 = other).

Receipt of income support during the last 12 months was used as a poverty measure (0 = no, 1 = yes). Income support is the last resort social assistance in Finland [40]. Participants were also asked whether they needed an interpreter in using the health and social care services, which was used as a measure for language skills in this domain (0 = no, 1 = yes).

The indicator of discrimination in health services is based on the question ‘Have you encountered the following situations in your daily life in Finland’, with one of the sub-questions ‘You are provided with worse service than others from a doctor in a health centre or hospital’ (0 = no, 1 = yes). Health information seeking practices were assessed by the question ‘If you or your close ones fall ill, where do you look for information about diseases, symptoms and treatment?’. Answers were grouped into the following categories: 1 = visit the local health centre or call them, 2 = from internet pages in Russian, 3 = other (including from internet pages in Finnish, friends and relatives, NGOs, other).

In Finland, accessing digital health services requires having a digital identification (e-ID) [41] and the item asking about having an e-ID that was used as one of the factors (0 = no, 1 = yes).

### Analysis

Multiple correspondence analysis (MCA) was performed using Burt’s approach as a dimension reduction procedure on the six circumstances that made it difficult to access the health services. The predicted row score of the first dimension was used as an outcome variable. MCA is a technique analogous to principal component analysis, specifically designed to analyse categorical variables [42]. MCA is preferred over factor analysis for the analysis of binary variables [43].

Three multiple ordinary least-squares linear regression (OLS) models were built for the MCA predicted score as an outcome variable. We ran regression analysis for two age groups: (M1) people 50–64 years of age (also referred to as the working age group) and (M2) people who were 65 years old or older (also referred to as the older group). The third model (M3) included both age groups. The three models included the same indicators and control variables, such as sociodemographic factors, migratory background, and indicators for information seeking and e-ID, except for the excluded employment status variable for the older group. The analyses were conducted using Stata 18. All models were estimated using the sampling weights accounting for non-response bias and survey design.

## Results

### Characteristics of the sample

Table 1 presents the characteristics of the sample. Approximately 60% of the research participants were between 50 and 64 years of age, and there were more men than women (57% and 43%). About half of the participants had obtained higher education in their country of origin. A higher proportion of working age participants had higher education than the older participants (52.7% and 48.1%) and had received some education in Finland (46.1% and 23.1%, respectively). A third of the sample was living in the capital region of Finland (33.8%). There were more people married or cohabiting among 50- to 64-year-olds than in the older age group (79.1% and 67.7%). A substantial proportion of working age participants were not in paid employment (33.1%), and close to half of all participants received income support (41.6%). Having Finnish citizenship was more common in the working age group than in the older participants (51.8% and 44.8%). The younger cohort expressed a lower need for an interpreter than the older cohort (36.1% and 46.5%) but had experienced more discrimination in health services (18.8% compared to 13.4%).

For the question ‘If you or your close ones fall ill, where do you look for information about diseases, symptoms and treatment?’, 57.4% of the working age group indicated that they called or visited the health centre, and 26.8% reported that they used internet pages in Russian, compared to 67.1% and 18.8% of 65 + participants in the respective categories. Only 67.9% of the entire sample and 53.8% of 65-year-olds and older had an e-ID at their disposal. This question had a sizable missing category, as 10% of the working age cohort and 18.7% of the older ones had left it unanswered. On average, the working age group had lived in Finland for 16 years, while the older participants had resided in Finland for 19 years.

**Table 1:** Characteristics of the study sample

	50–64 years old (%)	65 + years old (%)	All (%)
Age	60.3	39.7	100
Gender: Men	57.3	56.4	56.9
Education in home country			
General, none, or missing	4.7	7.8	5.9
Vocational	42.6	44.1	43.2
Higher	52.7	48.1	50.9
Have completed some education in Finland	46.1	23.1	37.0
Live in the capital region	33.1	35.0	33.8
Married or cohabiting	79.1	67.7	74.6
Employment status			
Employed	62.7	8.1	41.4
Pension	2.9	87.7	35.9
Other	34.5	4.3	22.7
Average or poorer self-rated health	53.6	69.3	59.8
Used health services more than twice last year	40.3	49.5	44.0
Received income support	35.3	51.8	41.6
Citizen of Finland	51.8	44.8	49.0
Length of stay in Finland in years (mean)	16.4	19.0	17.4
Length of stay in Finland in years (std)	8.7	9.3	9.1
Needs interpreter in health services	36.1	46.5	40.2
Experienced discrimination in health services	18.8	13.4	16.7
Health information sources			
Visiting health centre	57.4	67.1	61.2
Internet pages in Russian	26.8	18.8	23.6
Other	15.9	14.1	15.2
Having e-ID			
Has e-ID	77.2	53.9	67.9
e-ID missing category	10.0	18.7	13.4
Barriers to health services			
Long waiting lists	33.0	41.4	36.2
Poor transport connection	5.0	3.4	4.4
High price of services	19.5	22.5	20.6
Impolite or poor service	4.0	2.0	3.3
Language difficulties	25.4	36.8	29.6
Complicated structure	22.3	28.3	24.5

The most common barriers to health services were long waiting lists for services (36.2%), language difficulties (29.6%) and that the system was too complicated (24.5%) (see bottom of Table 1). A high price of health services was answered by 20.6% of the participants, while transportation and poor quality of service were less popular responses (4.4% and 3.3%, respectively). Each of the barriers except transportation and impolite or poor service were reported more often by the older participants.

### Composite index

**Table 2:** MCA results for each barrier

Barrier	Categories	Weights
Waiting time	Yes	0.162
	No	0.079
Transport	Yes	0.074
	No	0.003
High price	Yes	0.153
	No	0.034
Impolite or poor service	Yes	0.064
	No	0.002
Language difficulties	Yes	0.114
	No	0.043
Complicated structure	Yes	0.211
	No	0.061

**Fig. 1:**
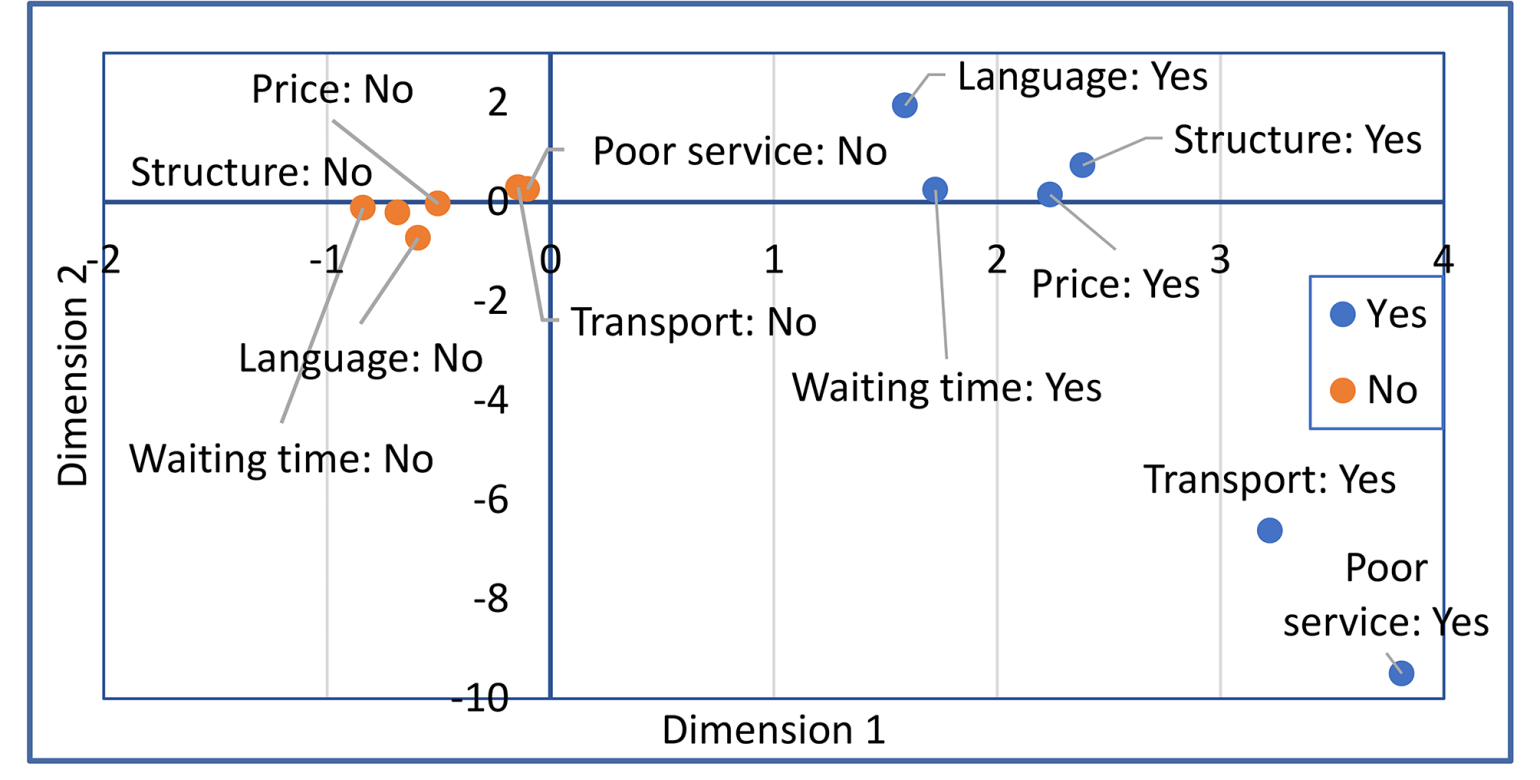
MCA barriers coordinate plot for dimensions 1 and 2

Table 2 lists all the variables underpinning the composite index score with categories and weights specifying the contribution of each variable to the principal dimension. The weights are identified from the first dimension of the MCA with iterative adjustment. Figure 1 presents the results of the MCA of the barriers to health services. The first dimension loaded most (highest inertia), explaining 91.8% of the total variance. Dimension 2 explained just 0.1%, which underlined the unidimensional nature of self-reported barriers. The distribution of points along Dimension 1 shows a clear pattern for those who selected ‘No’ and ‘Yes’, which are grouped together on the left and right sides of the chart, respectively. As a result, we predicted a row score of the first dimension that was used as an outcome variable in the regression models. This composite index of barriers ranged between − 1.4 and 4.8 and averaged 2.2, where a higher score indicates more barriers, and a negative score indicated having the fewest barriers.

### Regression analysis

Table 3 shows the results of the regression models adjusted for self-rated health and service use variables, the first model for the 50–64-year-old paticipants, the second for 65-year-old or older participants, and the third model for all participants.

Of the sociodemographic variables (RQ1), income support, employment status, region, and marital status were not associated with the barriers to health services in any model. Gender was associated with the outcome variable in the working age group and in the model including all ages; women were more likely to face barriers to health services. Having vocational education was associated with a higher prevalence of barriers in the total sample.

Regarding migration-related variables (RQ2), length of stay and Finnish citizenship were not associated with barriers to health services in any of the models. The need for interpreter and having experienced discrimination increased the likelihood of reporting barriers to health services in both age groups.

Our third RQ concerned information-seeking practices. The results showed that older participants who looked for health information online in Russian language instead of going to the health centre were more likely to face healthcare barriers. Having an e-ID was not associated with the healthcare barriers in any of the models. However, the category of missing value was associated with barriers to health services among older participants.

**Table 3:** OLS regression models with MCA predicted score as dependent variable

	50–64 years olds	65 + years olds	All participants
	Coefficient (S.E.)	Coefficient (S.E.)	Coefficient (S.E.)
Men	− 0.28 (0.10)**	− 0.04 (0.15)	− 0.21 (0.08)*
Education in home country: Vocational	0.41 (0.22)	0.28 (0.23)	0.37 (0.18)*
Education in home country: Higher	0.22 (0.22)	0.37 (0.23)	0.26 (0.17)
Have completed any education in Finland	0.07 (0.10)	0.24 (0.18)	0.10 (0.09)
Capital region	0.04 (0.11)	0.22 (0.16)	0.08 (0.10)
Married or cohabiting	− 0.09 (0.14)	− 0.15 (0.17)	− 0.09 (0.11)
Employment status: received pension	− 0.28 (0.22)	-	0.15 (0.11)
Employment status: other	0.05 (0.12)	-	0.00 (0.11)
Average or poorer self-rated health	0.15 (0.12)	0.38 (0.16)*	0.22 (0.10)*
Used health services more than twice last year	0.16 (0.12)	0.29 (0.14)*	0.21 (0.10)*
Receives income support	0.21 (0.13)	0.27 (0.17)	0.19 (0.10)
Citizen of Finland	0.08 (0.12)	− 0.06 (0.19)	0.04 (0.11)
Length of stay in Finland	0.00 (0.01)	0.01 (0.01)	0.00 (0.01)
Needs interpreter in health services	0.62 (0.16)***	0.54 (0.20)**	0.60 (0.13)***
Experienced discrimination in health services	0.71 (0.15)***	0.61 (0.23)**	0.72 (0.13)***
Health information sources: internet in Russian	0.18 (0.13)	0.37 (0.18)*	0.24 (0.11)*
Health information sources: other	0.03 (0.14)	0.10 (0.24)	0.04 (0.12)
Has e-ID	− 0.15 (0.15)	0.22 (0.17)	0.07 (0.11)
Has e-ID: missing category	0.11 (0.30)	0.63 (0.29)*	0.43 (0.22)*
N	511	301	791
Adjusted r-square	0.23	0.20	0.22

Statistical significance: *p <.05; **p <.01; ***p <.001.

## Discussion

The study examined the determinants of self-reported barriers to health services among Russian-speaking migrants 50 years of age or older in Finland. It contributes to our understanding of the systemic circumstances that the studied population reported as hindering their access to health services, drawing on representative survey data.

### RQ 1: sociodemographic factors

Most ***sociodemographic*** characteristics, such as education in the country of destination, region, marital status, employment status, and poverty, were not associated with the barriers to health services, which contradicts many previous studies, also studies in countries with universal health services provision [17, 23, 44, 45]. The findings of this study align with previous research conducted in Nordic countries, which highlights that women tend to face more disadvantages in accessing and using health services [46, 47]. This is consistent with studies conducted in contexts other than the Nordics [4, 48]. However, in our study, a gender difference was not found in the older age group, which means that we cannot extend these findings to migrants 65 years and older.

### RQ 2: migration-related factors

Of **migration-related factors**, the need for an interpreter and experienced discrimination were associated with an increased likelihood of reporting barriers to health services in both 50–64 and over 65 year-olds. Previous studies on younger working-age migrant populations have found that those who experience discrimination are more likely to face barriers to health services [4, 22]. In the same way, the need for language interpreters in health services has constituted a significant access barrier for migrants in other studies [1, 22]. Barriers related to language skills and discrimination have been found across different country contexts, in North American, European as well as Finnish studies [2, 8, 24]. Other factors related to individual migration histories, such as length of stay and citizenship status, were not associated with the outcome variable, which is dissimilar to previous observations of migrants’ health service access and use [11, 18].

As previous findings are limited to migrants of working age, the results of this study extend them to older migrants as well, meaning that discrimination and interpreter need increase the likelihood of facing health service barriers among migrants 65 years of age and older. This is highly problematic, considering the heightened healthcare needs in older age. Additionally, with advancing age, problems with cognition and memory can exacerbate access barriers related to language and cultural differences.

In Finland, intriguingly, interpreters are formally provided at no cost for individuals who do not speak the local languages, i.e., Finnish, or Swedish [49]. However, their accessibility remains limited, particularly in cases of acute care, due to the requirement of pre-ordering interpreters. Previous research has outlined substantial challenges associated with interpreter services, particularly concerning privacy and the quality of interpretation [50, 51]. A recent population-based study conducted in Finland showed that only approximately 10% of working-age migrants from Russia or other former Soviet Union countries utilised interpreter services within health settings [34]. The current study indicated that among Russian-speaking migrants aged 50 years and above, 40% acknowledged the need for interpreter assistance. While making direct comparisons between these studies is difficult, the substantial gap between the demonstrated need for interpreter services and their actual utilisation potentially sheds light on the association between the need for interpreter and the increased barriers to health services found in the present study. This circumstance could imply that a substantial portion of Russian-speaking adults 50 years of age or older might face the necessity of postponing seeking healthcare, attempting to navigate healthcare services without the aid of an official interpreter and seek help, for example from family and friends. Such a situation could potentially jeopardise the standard of care received, consequently giving rise to adverse outcomes concerning the patients’ health. In addition, there is an issue of right to privacy: the person may not be comfortable of sharing their personal and often sensitive health information even with their spouse or their underage or adult children or grandchildren [52].

As previous studies have showed that language barriers contribute to perceived unmet needs of migrant populations in Finland [2, 3], in future research, it would be important to study the different strategies of overcoming the language barriers and the need of interpreter specifically.

### RQ 3: health information seeking

Most public as well as private healthcare providers have implemented digital systems, e.g. for scheduling appointments, accessing personal health records, viewing and renewing prescriptions, engaging in service chats, and conducting remote consultations, with the majority of these services necessitating the use of an e-ID. It has been shown that obtaining an e-ID is not straightforward for migrants in Finland [28]. Approximately 77% of the 50–64 year old and 54% of over 64 year old participants of this study had an e-ID in their use. In contrast, among the general population, 98% of the working-age population have an e-ID, while 89% of individuals aged 55–74 and 57% of those older than 74 years possess it [27, 53]. Unlike previous findings highlighting the importance of the e-ID for overall access to and use of health services [26, 27], the results of this study did not show any association between having an electronic ID and the studied outcome variable. However, the missing value category was associated with the prevalence of barriers to health services. This may indicate that those who did not understand the meaning of electronic identification and thus presumably did not have access to digital health services were also more likely to face barriers.

In the Nordic context, it has been previously found that using the internet as a source of health information and an ability to look for health information online significantly improves access to health services [54, 55]. The analysis of this study demonstrated that using the Russian-language internet pages as the primary source of health information, meaning instead of going to or calling the local health centre or other sources, was associated with reporting more barriers to health services. This could indicate the concerns expressed in earlier research regarding the dependence on health information obtained from online sources as the quality of medical information is variable [56]. This situation can have a detrimental effect on patient‒physician relationship, and may result in patients experiencing distress, and increasing their inclination towards self-treatment [56].

Furthermore, difficulties in accessing healthcare may prompt individuals to seek health information from unreliable sources or travel abroad for medical care [57]. Previous study showed that in 2019 a quarter of midlife and older Russian-speaking migrants had used healthcare services outside Finland in the last 12 months [58]. While opting for transnational healthcare can offer opportunities for receiving culturally preferred styles of care, it can also bring challenges such as potential double medication, and delays or discontinuity in the provision of care. Similar to looking up information from foreign websites, choosing to seek care abroad may result in receiving health information conflicting with the official health recommendations provided by the local healthcare professionals.

### RQ 4: age differences

Our findings indicate that especially the older age groups of Russian-speaking participants have difficulties in accessing healthcare. In addition to long waiting lists, the most common barriers included language difficulties, high price of services, and a complicated structure of the Finnish healthcare system. Previous studies addressing access barriers for older adults in Finland in general have highlighted factors like high costs and geographical distance to healthcare facilities [33]. Although our study participants noted the high price of health services, issues such as transportation and poor service quality were less frequently mentioned compared to obstacles associated with language and the difficultly of the system. This may be because migrants often live in the bigger cities, where services are still rather easy to reach.

Differences between age groups in the regression models were mostly marginal, but female gender was more strongly associated with barriers to health services in the 50–64 year-old cohort. This is likely because women in that age group were using healthcare services the most of all age and gender categories.

In addition, information-related factors had a stronger relationship with barriers among older participants. As higher incidence of most barriers was observed in older age cohort, relying on online health information in participant’s own language may represent a way to compensate for the lack of access to formal healthcare.

## Conclusions

### Strengths and limitations

There are several strengths in this study. First, our unique data allowed us to carry out a representative study of a particularly hard-to-reach group, older migrants. Second, the question on language interpreter needs in healthcare services allowed us to explore the language-related challenges in this specific domain. Third, our versatile data allowed us to separate the self-reported barriers from the social determinants of access to health services. Fourth, MCA allowed us to create a composite index with categorical variables, as the results uncovered the unidimensional nature of the self-reported barriers. Additionally, it enabled the analysis of multiple barriers as a continuous variable instead of dichotomous outcomes.

The study also has some limitations. There was no comprehensive measurement of health literacy that previous articles have showed to be important for accessing health services [4, 25]. Furthermore, the study of Russian speakers does not represent all migrant groups in Finland, as previous research has pointed to vast differences across migrant groups [34]. As Russian speakers are the largest foreign language group in Finland, are highly educated, resided in Finland on average for a long time, and are considered to be rather well integrated into the Finnish system, they may in fact face fewer barriers to healthcare than other people with a migratory background.

### Policy implications

This study has important policy implications. It is imperative for policymakers to tackle the challenges posed by waiting lists and language-related barriers regarding accessing health services. One potential approach is to offer cultural training and resources to medical personnel, alongside enhancing the availability of interpreter services. Furthermore, presenting information in various languages can help patients in navigating the route to healthcare, potentially diminishing dependence on less reliable internet sources. Policymakers must prioritise these measures to safeguard equitable access to health services for every individual, irrespective of their local language proficiency.

### Conclusion

The results of this study indicate that Russian-speaking migrants 50 years of age or older face multiple barriers to health services in Finland. We also found that determinants related to a migratory background and information-seeking practices are strongly associated with reporting barriers to health services. Given that the population– including the migrant population– is ageing, and that healthcare needs increase with age, addressing these issues becomes crucial, necessitating improved access to health services and to health information for migrants in Finland.

## Data Availability

The datasets used and analysed during the current study are available from the corresponding author on reasonable request.
